# Secretory carrier membrane protein 5 is an autophagy inhibitor that promotes the secretion of α-synuclein via exosome

**DOI:** 10.1371/journal.pone.0180892

**Published:** 2017-07-11

**Authors:** Yi Yang, Meiling Qin, Puhua Bao, Wangchao Xu, Jin Xu

**Affiliations:** 1 State Key Laboratory of Neuroscience, Institute of Neuroscience, Chinese Academy of Sciences, Shanghai, China; 2 University of Chinese Academy of Sciences, Shanghai, China; Univerzitet u Beogradu, SERBIA

## Abstract

Autophagy-lysosomal pathway is a cellular protective system to remove aggregated proteins and damaged organelles. Meanwhile, exosome secretion has emerged as a mode to selectively clear the neurotoxic proteins, such as α-synuclein. Mounting evidence suggests that these two cellular processes are coordinated to facilitate the clearance of toxic cellular waste; however the regulators for the transition between these two processes are unclear. Here we show that SCAMP5, a secretory carrier membrane protein significantly induced in the brains of Huntington's disease patients, is quickly and transiently induced by protein stress and autophagic stimulation, and is regulated by the master autophagy transcriptional regulator TFEB. Ironically, SCAMP5 inhibits autophagy flux by blocking the fusion of autophagosomes and lysosomes. Although autophagy is blocked, SCAMP5 does not cause significant protein aggregation in cells. Instead, it promotes the Golgi fragmentation and stimulates the unconventional secretion of the co-localizing α-synuclein via exosome as an exosome component. Therefore, we have identified SCAMP5 as a novel coordinator of autophagy and exosome secretion, which is induced upon protein stress to channel the efficient clearance of toxic proteins via the exosomes rather than autophagy-lysosomal pathway.

## Introduction

A common theme for the development of neurodegenerative diseases is the propensity of a number of proteins, such as α-synuclein, Huntingtin (HTT), and Tau, to misfold and form insoluble aggregates [1, 2]. If not efficiently cleared, these protein aggregates will impair neuronal functions, and eventually cause cell death [3–5]. The main cellular pathways to sustain the protein homeostasis are autophagy and ubiquitin proteasome, two intertwining machineries that facilitate the degradation of misfolded proteins, aggregates and even damaged organelles [6]. Consequently, agents that stimulate protein clearance, such as Rapamycin, have shown neuroprotective activities in various models of neurodegeneration [7, 8].

Recently, mounting evidence has suggested that those aggregation-prone neurotoxic proteins, such as α-synuclein, HTT, Tau, can also spread among various brain regions with time [9–11]. This theory may account for the worsening neuropathology in multiple brain regions in the late stages of various neurodegenerative diseases[12, 13]. Furthermore, secretion of misfolded proteins upon protein stress could be actively regulated and represent an adaptation to proteasome dysfunction [14]. Similarly, when autophagy is pharmacologically inhibited, α-synuclein and TDP43 can be secreted through exosomes[15, 16]. Exosomes are 30-100nm vesicles, form within multivesicular body (MVB) and facilitate the transmission of RNA and proteins between cells [17]. However, the regulatory machineries that facilitate protein accumulation, secretion and uptake are not well characterized.

Secretory carrier membrane protein 5 (SCAMP5) is one of the five members of the SCAMP family proteins, which are proteins regulating membrane trafficking [18, 19]. While SCAMP 1-4 are ubiquitously co-expressed, SCAMP5 is enriched in the brain and expressed robustly at the synapse late in brain development [18]. Recently, loss of SCAMP5 has been implicated as a potential genetic risk for autism [20]. Furthermore, silencing of SCAMP5 expression reduces the abundance of synaptic vesicles and impairs endocytosis [21]. On the other hand, SCAMP5 accumulates in the striatum of Huntington's disease patients and could aggravate mutant HTT aggregation [22], although the underlying mechanism was not clear. In this study, we investigated the regulation and function of SCAMP5 during protein stress and found SCAMP5 as an interesting regulator of protein homeostasis that relieves protein stress by affecting both autophagy and secretion. Paradoxically, this protein clearance mechanism during stress may promote the propagation of α-synuclein.

## Materials and methods

### Plasmids and siRNA

Expression plasmids of FLAG-tagged *Homo sapiens* SCAMP5, SCAMP1, and TFEB were generated by PCR using human cDNA library and subcloned into the pcDNA3+based vector (Invitrogen). EGFP-α-synuclein-WT and EGFP-α-synuclein-A53T were generated by subcloning into the EGFP-C1 vector and mutagenesis. Tandem Fluorescent-Tagged LC3 (tf-LC3 or mRFP-GFP-LC3) was kindly provided by Dr. Yoshimori [23]. Luciferase reporter constructs pGL3-pSCAMP5 was generated by PCR of the predicted human SCAMP5 promoter (~3400bp upstream of SCAMP5 mRNA) using human genomic DNA library and cloned into pGL3-based vector (Promega). Lentivirus Lv-pSYN-Flag-SCAMP5 was generated by subcloning of SCAMP5 into the lv-fhSYN-eNpHR3.0-E (minus 2a-GFP) vector, and the lentivirus was generated in HEK-293T cells with Pspax+pMD2.G coating plasmids. The human SCAMP5 and TFEB siRNA constructs were synthesized at Genepharma with the following sequences: SCAMP5-542(sense, 5’ CCCAUUUACAAGGCCUUCATT; antisense, 5’UGAAGGCCUUGUAAAUGGGTT); SCAMP5-771(sense, 5’ CCCUCAGCAUGGUUCAUAATT; antisense, UUAUGAACCAUGCUGAGGGTT); SCAMP5-1101(sense, 5’ GGACCAGAGUUAUAUAUAUTT; antisense, AUAUAUAUAACUCUGGUCCTT); TFEB-870(sense, AGACGAAGGUUCAACAUCATT; antisense, UGAUGUUGAACCUUCGUCUTT); TFEB-981(sense, UACAUCCGGAGGAUGCAGATT; antisense, UCUGCAUCCUCCGGAUGUATT).

### Cell culture, transfection, and stable cell lines

SH-SY5Y[24] and HEK293T[25] cells were obtained from American type culture collection (ATCC). Cell lines were cultured at 37°C in 5% CO_2_ in DMEM medium (Invitrogen) supplemented with 10% fetal bovine serum (Invitrogen) and antibiotics (HyClone).

Primary neuronal cultures were collected from the cortex of P0 rats, and cultured at 37°C in 5% CO_2_ in Neurobasal medium (Invitrogen) supplemented with B-27 and GlutaMAX (Invitrogen). This procedure was approved and performed in accordance with the regulations by the Ethics and Animal Care and Use Committee of the Institute of Neuroscience, Shanghai Institutes for Biological Sciences (Permit Number: NA-011-2016).

Cell lines were transfected using Lipofectamine 2000 reagent (Invitrogen) for over-expression or Lipofectamine RNAiMAX (Invitrogen) for gene silencing. For lentiviral transduction, day 5 primary neuronal cultures were incubated with lentivirus for 24 hours, then cultured in fresh neuronal medium for another 48 hours before harvest. To generate SH-SY5Y cells stably expressing EGFP-α-synuclein-WT or EGFP-α-synuclein-A53T, plasmids (with neomycin selective marker) were transfected into SH-SY5Y cells. Two days later, the transfected cells were selected by adding 1mg/ml G418 in culture medium, followed by fresh medium change every a few days to remove non-adherent cells as needed. The stably-transfected cells were then plated in 96-wel plate at single cell per well density for culturing to further expand individual colonies and selected for the ones with robust expression. The selected cells were maintained in 400μg/ml G418.

### Antibodies and drugs

The primary antibodies used are: rabbit anti-SCAMP5 (ab3432, Abcam;1:500 for WB); rabbit anti-LC3B (L7543, Sigma-Aldrich; 1:3000 for WB); mouse anti-p62/SQSTM1 (ab56416, Abcam; 1:2000 for WB); rabbit anti-TFEB (13372-1-AP, Proteintech; 1:1000 for WB); mouse anti-α-synuclein (610787, BD Biosciences; 1:1000 for WB); rabbit anti-huntingtin (5656P, Cell signaling; 1:1000 for WB); mouse anti-GM130 (610822, BD Biosciences; 1:300 for IF); mouse anti-Giantin (ab37266, Abcam; 1:300 for IF); rabbit anti-ERGIC53 (sc-66880, Santa Cruz; 1:100 for IF); rabbit anti-Calnexin (ab22595, Abcam; 1:2000 for WB); mouse anti-CD63 (ab8219, Abcam; 1:1000 for WB); mouse anti-DYKDDDDK-Tag (FLAG) (M20008, Abmart; 1:3000 for WB; 1:300 for IF; 1:200 for IP); goat anti-DYKDDDDK-Tag (FLAG) (NB600-344, NOVUS; 1:100 for IF); mouse anti-HA-Tag (M20003, Abmart; 1:200 for IP); mouse anti-GFP-Tag (M20004, Abmart; 1:2000 for WB); mouse anti-β-tubulin (M20005, Abmart; 1:3000 for WB); mouse anti-β-actin (M30002, Abmart; 1:2000 for WB).

The drugs used are: Rapamycin (553210, Merck, 100nM); Bafilomycin A1 (196000, Merck, 10-50nM); MG132 (474790, Merck, 3-5μM); Cycloheximide (C7698, sigma, 100μg/ml); Tetanus toxic (T3194. Sigma-Aldrich, 2nM); BrefeldinA (S1536, Beyotime, 2μM).

### Luciferase assay and qPCR

For Luciferase assay, cells were transfected with empty vector or TFEB, together with firefly luciferase reporter pGL3-pS5 and renilla(as internal control, Luc:Ren = 30:1). 48 hours later, the cells were lysed for dual-luciferase bioluminescence assay analysis (E1910, Promega). For each experiment, samples were analyzed in triplicates. For Realtime PCR (qPCR), total RNAs were extracted from cells using Trizol. The full-length cDNA library was constructed by reverse transcription PCR using PrimeScriptTM RT Master Mix Perfect Realtime (#RR036A, Takara). qPCR was performed using iQ TM SYBR@ Green Supermix (1708882, BioRad) with BioRad CFX Connect real time PCR system. The specific primer sets are as follow: SCAMP5(F:5'TCTGGATGTTGAACAGCGTCA, R: 5'AAACCAGCAGACGTAGGAGC); actin(F: 5'GGCTACAGCTTCACCACCAC, R: 5'GAGTACTTGCGCTCAGGAGG); TFEB(F: 5'GGTGTTGAAGGTGCAGTCCT, R: 5'GTGGGCAGCAAACTTGTTCC).

### Immunoblotting and immunoprecipitation

For standard SDS-PAGE, cells were lysed with RIPA buffer (50mM Tris pH7.4, 150mM NaCl, 50mM NaF, 1mM EDTA, 1% Triton X-100, 0.5% deoxycholate, 0.1% SDS, adding protease inhibitor cocktail (Roche)) for 30 min, and centrifuged at 13,000rpm for 15 min at 4°C. The supernatant was kept for RIPA soluble fraction. The pellet was resuspended with RIPA buffer again for washing, and centrifuged at 13,000rpm for 15 min. The final pellet was lysed with 8M Urea (8M urea, 50 mM Tris-HCl pH 8.0, 1 mM EDTA, 100mM NaCl) as RIPA insoluble fraction. The cytoplasmic and nuclear fraction were extracted using NE-PER® Nuclear and Cytoplasmic Extraction Reagents (78833, Thermo scientific). For native PAGE, the cells were lysed with 0.2% NP-40 Lysis buffer (20 mM HEPES, pH 7.2, 0.32 M sucrose, 5mM MgCl2, 2mM ATP, 0.2% NP-40 and protease inhibitors), loaded with 2x native sample buffer (62.5mM Tris-HCL, pH6.8, 40% glycerol, 0.01% bromophenol blue), and run native PAGE (-SDS) with Mini-PROTEAN TGX precast gel (#456-1095 BIO-RAD).

The procedure for immunoprecipitation was described previously [26]. For the analysis of ubiquitination of SCAMP5, HEK-293T cells were transfected with HA-Ubiquitin, Flag-SCAMP5, or both for 24 hours, then lysed in RIPA buffer supplemented with protease inhibitors cocktail (Roche) and 20mM N-ethylmaleimide (E3876, Sigma). The cell lysates were pre-cleared with mouse IgG and protein A/G sepharose (A10001, Abmart), and incubated with mouse anti-Flag antibodies for 12 hours and then added protein A/G sepharose for another 4 hours at 4°C with gentle inversion mixing. The beads were washed four times with lysis buffer, and eluted by Elution buffer (0.2% SDS, 0.1% Tween20, 50mM Tris-HCl pH 8.0) followed by SDS-PAGE analysis.

### Immunofluorescence and imaging

For immunofluorescence, SH-SY5Y cells were cultured on glass coverslips, and for HEK-293T cells the coverslips were coated with Poly-L-lysine hydrobromide (SIGMA, P6282). The cells were fixed with 4% paraformaldehyde for 15 min, permeabilized with 0.25% TritonX-100/PBS for 10 min, blocked with 3% BSA/PBS (Blocking buffer) for 30 min. Then specific primary antibodies (diluted in blocking buffer) were applied for 12 hours at 4°C, followed by Alexa Fluor conjugated secondary antibodies accordingly (Donkey anti Mouse/Rabbit/Goat-488/546/647, Invitrogen; diluted 1:500 in blocking buffer) for 2 hours at RT, and mounted with a drop of Vectashield mounting solution(94010, Vector Laboratories, Inc.). Images were taken with an inverted laser scanning confocal microscope (Nikon A1R) with 60X TIRF oil immersion lens.

### MTS assay

SH-SY5Y cells were digested and counted, then resuspended in fresh medium to make a final concentration of 10^6^ cells/ml. 100μl of cells and 20μl of the CellTiter 96® AQueous One Solution Reagent (Promega, G3580) were mixed and added to each culture well of 96-well plate. For each experiment, samples were analyzed in triplicates. The plate was then incubated for 2 hours at 37°C in a humidified, 5% CO_2_ atmosphere and the absorbance was recorded at 490nm with a 96-well plate reader.

### Exosome purification

The procedure for exosome isolation was modified from previous protocol [27]. To remove the exosomes from FBS, DMEM and FBS were first mixed at 1:1 (vol;vol), and centrifuged at 100,000g for 16 h at 4°C. The resulting exosome-free DMEM-FBS mixture was further diluted in DMEM to make the DMEM with 10% FBS as cell growth medium. To isolate exosomes from culture medium, conditioned medium from cell culture was collected and centrifuged for 5 min at 1,200g followed by 20 min at 13,000g to exclude cell debris. Then the cell medium was incubated with Total Exosome Isolation Reagent (from cell culture media) (4478359, Invitrogen) at 4°C overnight, and centrifuged at 10000g for 1 hour. Exosome pellets were lysed with RIPA buffer or native lysis buffer (for CD63), or resuspended in Phosphate Buffer for electron microscopy.

### Electron microscopy

Aliquots of exosome suspensions were dispensed onto sheets of Parafilm in a humidified petri dish and the vesicles were deposited on carbon-coated grid (300-mesh) for 3 min. Subsequently, the grid was negatively stained with 1% uranyl acetate for 3min and excess stain was blotted off. After drying grids were analyzed with a transmission electron microscopy (JEM-1230, JEOL, 80kV).

### Statistical analyses

The band intensity in immunoblots was determined by BIO-RAD Quantity One software. All experiments were performed at least in triplicate, and the data are presented as the mean ±S.E.M. The statistical significance was analyzed using student t-test for experiments with only two groups. Analysis of variance (ANOVA) was performed for all experiments with more than two groups, and if statistical significance (P < 0.05) was achieved, we performed post hoc analysis to account for multiple comparisons (***, P<0.001, **, P<0.01; *, P<0.05).

## Results

### SCAMP5 is transiently up-regulated in response to protein stress and autophagic signals

**Fig 1:**
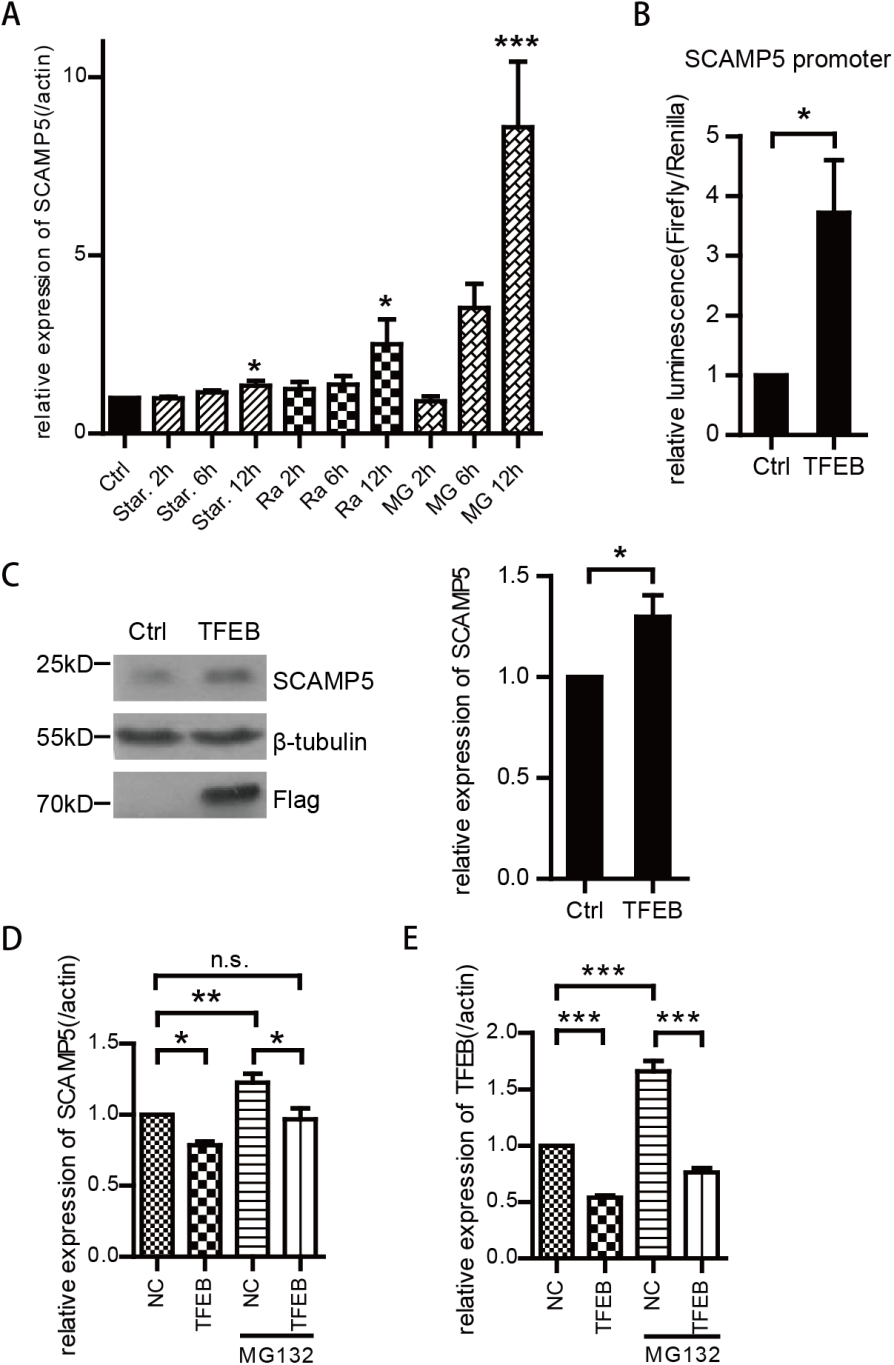
SCAMP5 is induced by autophagy regulator TFEB. (A) Real-time PCR results showing increased SCAMP5 level in primary neuronal cultures treated with Rapamycin(100nM), MG132(3μM) or starvation. Values were normalized to control group as relative expression with respect to endogenous control gene actin (mean ±S.E.M.; n = 3; *p<0.05). (B) Dual-luciferase assay showing that TFEB activates from the SCAMP5 promoter. SH-SY5Y cells were transfected with either an empty vector pcDNA3 or Flag-TFEB and a firefly luciferase reporter directed by the human SCAMP5 promoter. Renilla-luciferase reporter was co-transfected as transfection efficiency control. The relative luminescence of Firefly/Renilla was examined 48h later (mean ±S.E.M.; n = 6; *p<0.05). (C) Immunoblots showing increased SCAMP5 level in SH-SY5Y cells transfected with Flag-TFEB (left panel). Right panel shows the quantification of SCAMP5/β-tubulin expression in the immunoblots of three independent experiments (mean ±S.E.M.; n = 3; *p<0.05). (D) The expression of SCAMP5 during autophagy response in TFEB-silenced cells. SH-SY5Y cells were transfected with siRNA against TFEB (TFEB) or scrambled non-specific siRNA as control (NC), and exposed to MG132 for 6 hour before harvest for analysis by real-time PCR. The silencing of the TFEB in SH-SY5Y cells was confirmed in (E) The expression of TFEB and SCAMP5 were normalized to actin and then to the control (mean ±S.E.M.; n = 3; *p<0.05, **p<0.01, ***p<0.001).

SCAMP5 is up-regulated in the striatum of patients with HD [22]. To investigate the mechanism of SCAMP5 induction, we first tested whether the mRNA expression of SCAMP5 was induced by regulators of protein degradation and stress pathways. Although at different efficiency, the SCAMP5 mRNA could be induced by autophagy inducer starvation, Rapamycin, and proteasome inhibitor MG132 (Fig 1A). As autophagy-lysosomal pathway is activated under all those stress conditions, we examined whether SCAMP5 could be activated by the master lysosomal transcriptional regulator TFEB. Using the luciferase reporter directed by the human SCAMP5 promoter, we found that TFEB could activate SCAMP5 promoter (Fig 1B). The induction of SCAMP5 by TFEB was also confirmed at the protein level (Fig 1C). Next, we used siRNA to knock down TFEB in SH-SY5Y cells, and found decreased SCAMP5 mRNA level in these cells. When cells were treated with MG132, we found increased level of both TFEB and SCAMP5. Knocking down of TFEB at the same time diminished the induction of SCAMP5 by MG132(Fig 1D and 1E). Therefore, autophagy-related stresses induce SCAMP5 through the induction of TFEB.

**Fig 2:**
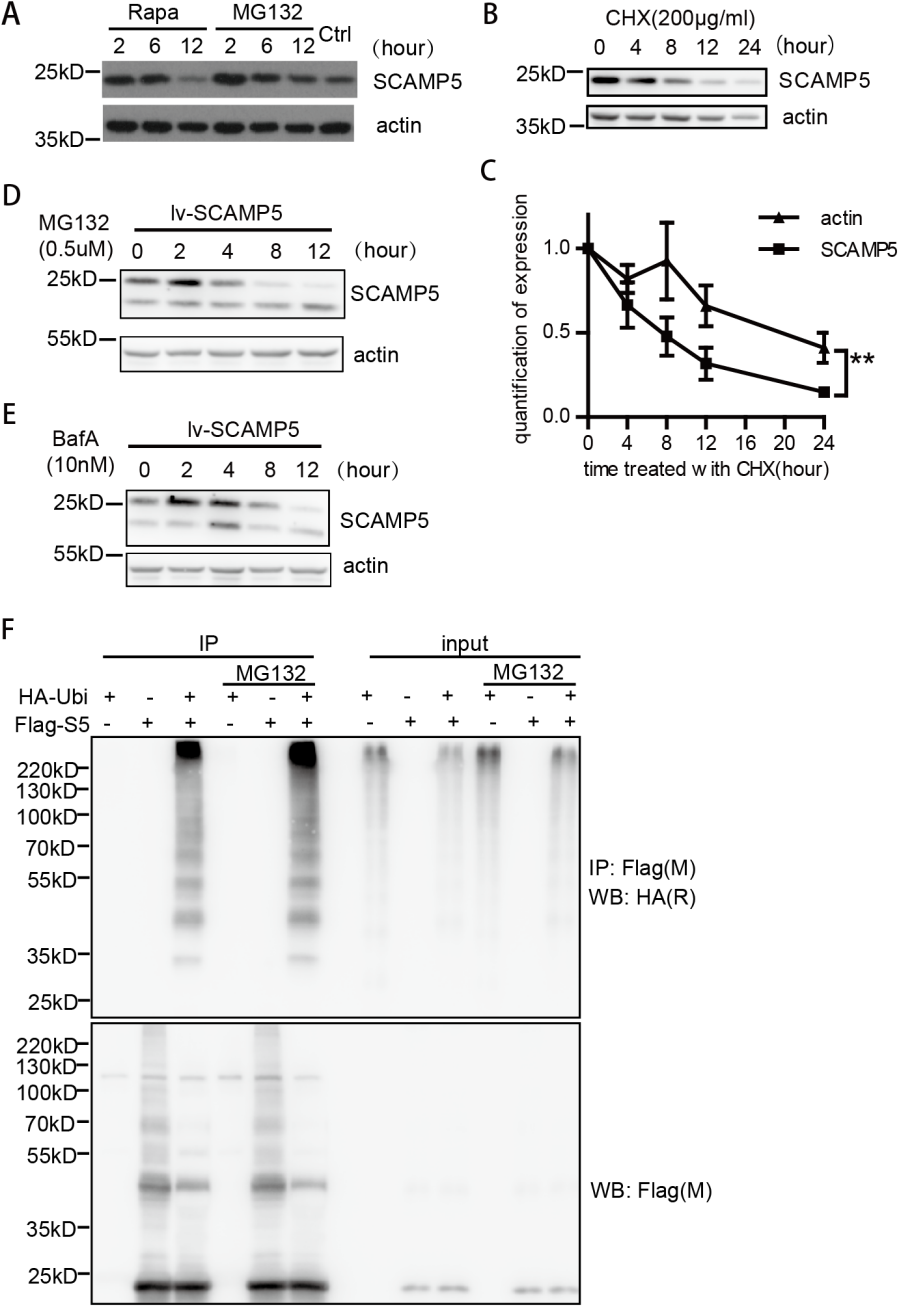
SCAMP5 is ubiquitinated and can be degraded by the proteasome or autophagy pathway. (A) Immunoblots showing quickly increased SCAMP5 in primary neuronal cultures treated with Rapamycin(100nM) or MG132(3μM). Representative results from four independent experiments with similar conclusions were shown. (B) Immunoblots and the quantification (C) showing that SCAMP5 degrades much quicker than β-actin. Primary neuronal cultures were treated with protein synthesis inhibitor Cycloheximide over a timecourse of 24 hours (mean ±S.E.M.; n = 3; **p<0.01). (D) Representative immunoblots showing that proteasome inhibitor MG132 causes a transient increase of exogenous SCAMP5. Primary neuronal cultures were infected with lentiviral vector expressing neuronal specific SCAMP5, and harvested 72 hours later. MG132 was added 2 to 12 hours before harvest. The results represent three independent experiments with similar results. (E) Representative immunoblots showing that autophagy inhibitor BafilomycinA1 causes a transient increase of exogenous SCAMP5 in primary neuronal cultures expressing lenti-viral delivered SCAMP5 as in (D). BafilomycinA1 was added 2 to 12 hours before harvest. The results represent three independent experiments with similar results. (F) SCAMP5 is ubiquitinated as shown by immunoprecipitation. HEK-293T cells were transfected with HA-Ubiquitin, Flag-SCAMP5, or both for 24 hours. One group was treated with MG132 for 6 hours before harvest. The cells were lysed with RIPA buffer and immunoprecipitated using anti-Flag antibody, then immunoblotted with anti-HA antibody.

Upon the examination of SCAMP5 protein level in primary neuronal culture exposed to Rapamycin and MG132, we found that the protein level of SCAMP5 was quickly and transiently increased (Fig 2A). We treated cells with protein synthesis inhibitor cycloheximide (CHX), and found that the protein stability of SCAMP5 was significantly lower than that of actin, indicating that SCAMP5 is quickly turned over (Fig 2B and 2C). Furthermore, virally-transduced exogenous SCAMP5 showed similar quick induction and degradation as the endogenous SCAMP5 when cells were treated with MG132, suggesting that even though SCAMP5 can be transcriptionally induced by autophagy signaling (Fig 1), the expression of SCAMP5 is also regulated at the protein level (Fig 2D). Although proteasome inhibitor MG132 caused accumulation of SCAMP5, it did not completely block the degradation of SCAMP5 at the end of treatment. Thus, we evaluated whether SCAMP5 was also degraded by the autophagy pathway. Indeed, Bafilomycin A1, an inhibitor that blocks autophagic degradation, also transiently increased the level of SCAMP5 (Fig 2E). Protein ubiquitination is a post-translational modification to regulate protein degradation by both proteasome and autophagy-mediated pathways [28]. Using immunoprecipitation assays, we found that SCAMP5 was indeed a substrate of ubiquitination, and the poly-ubiquitination of SCAMP5 was further increased when MG132 was added. (Fig 2F), confirming that the quick accumulation of SCAMP5 is due to degradation blockage. Once accumulated, both the proteasome and autophagy pathways contribute to the quick clearance of SCAMP5.

### SCAMP5 inhibited the autolysosome formation

**Fig 3:**
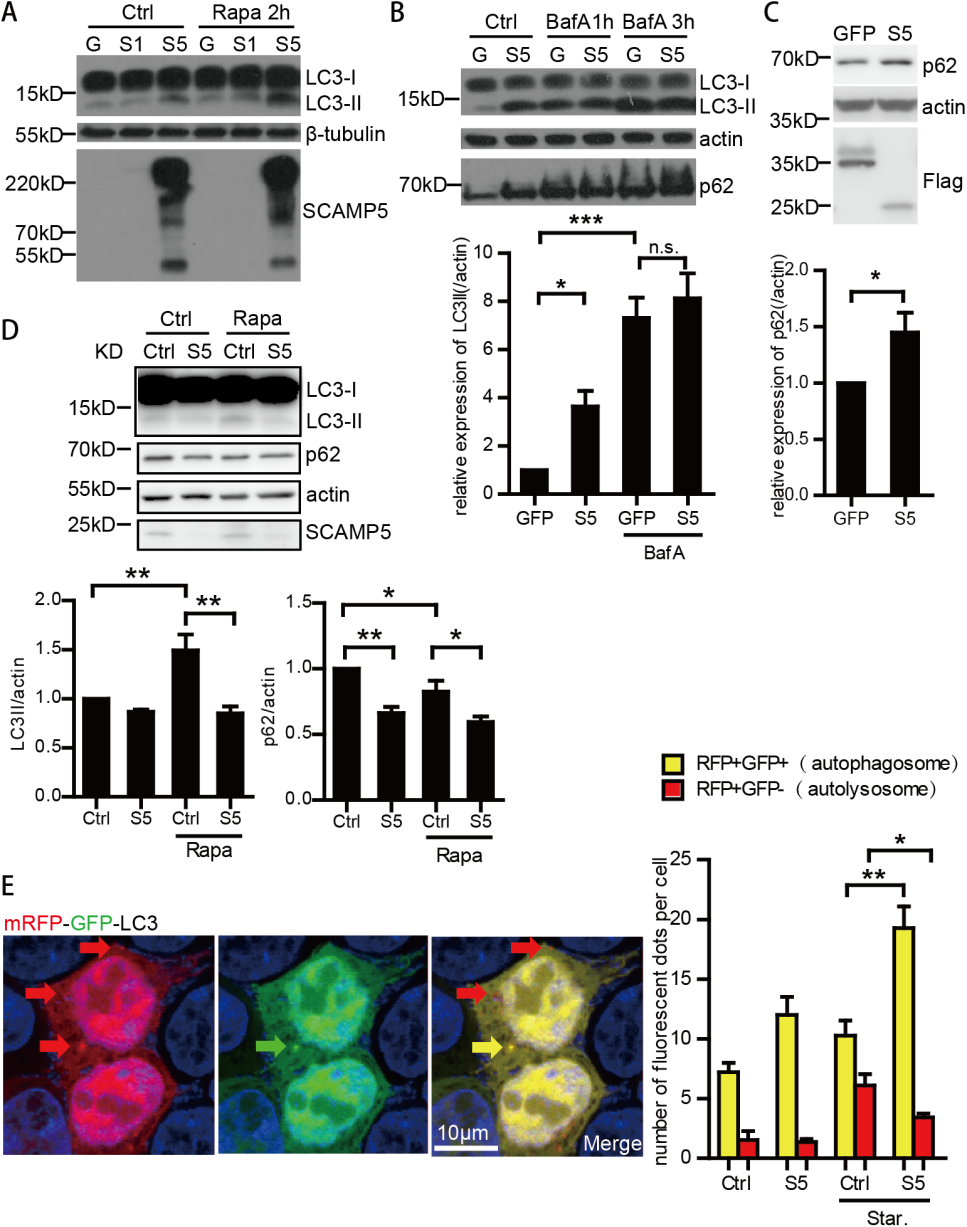
SCAMP5 blocks autophagy flux by inhibiting the fusion of autophagosomes and lysosomes. (A) Immunoblots showing increased LC3-II in SCAMP5 over-expressed cells. HEK-293T were transfected with Flag-GFP, Flag-SCAMP1 or Flag-SCAMP5, and harvest 48 hours later. Autophagy inducer Rapamycin (100nM) was added to culture medium for 2 hours before harvest. In SDS-PAGE, the appeared molecular weight of SCAMP5 was about 25kD when the sample was not boiled. Large aggregated bands would form when the sample was boiled, probably due to its property as a membrane protein. (B) HEK-293T cells were transfected with Flag-GFP or Flag-SCAMP5 and harvest 48 hours later. Autophagy blocker Bafilomycin A1 (50nM) was added to culture medium for indicated time before harvest. The protein level of LC3 and p62 was examined by immunoblotting (upper panel). Lower panel shows the quantification of LC3II/actin in three independent experiments, BafilomycinA1 was applied for 3 hours (mean ±S.E.M.; n = 5; *p<0.05, ***p<0.001). (C) Immunoblots showing increased p62 in cells over-expressing SCAMP5. SH-SY5Y cells were transfected with Flag-GFP or Flag-SCAMP5. Lower panel showed the quantification of three independent experiments (mean ±S.E.M.; n = 3; *p<0.05). (D) Immunoblots (upper panel) and quantification (lower panel) of the effect of SCAMP5 knockdown on LC3II and p62 in the absence or the presence of Rapamycin (mean ±S.E.M.; n = 4 independent experiments; *p<0.05, **p<0.01). (E) Immunofluorescent results of mRFP-GFP-LC3 showing that SCAMP5 blocks the fusion of autophagosome and lysosome. HEK-293T cells were transfected with autophagy reporter mRFP-GFP-LC3 and either empty vector or Flag-SCAMP5, one group was treated with FBS free DMEM medium (starvation) for two hours before harvest. Immunofluorescent was performed 24 hours later using mouse anti-Flag primary antibody and donkey anti mouse Alexa Fluor 647 secondary antibody. Confocal microscopy examination confirmed that all the mRFP-GFP-LC3 transfected cells were Flag-SCAMP5 positive(data not show). The acid-sensitive green fluorescence is lost when autophagosomes fuse with lysosomes as shown in the representative image (left panel). The quantification of the number of RFP+GFP+ autophagosome and RFP+GFP- autolysosome was carried out double-blindly in more than 300 cells(mean ±S.E.M.; n = 3; *p≤0.05, **p<0.01) (right panel).

As a prior report suggested that SCAMP5 caused the accumulation of mutant HTT [22], which is known to be regulated by autophagy [29], we explored the effect of SCAMP5 on autophagy. We transfected HEK-293T cells with Flag-tagged SCAMP5, SCAMP1 or GFP, then examined the level of LC3-II, a well characterized indicator of autophagy, via immunoblotting. SCAMP5, but not a closely related SCAMP1, caused the accumulation of LC3-II (Fig 3A). Rapamycin, an autophagy inducer, further augmented the accumulation of LC3-II caused by SCAMP5 (Fig 3A). As LC3II could only represent the amount of autophagosomes in cell, to examine whether the increase of LC3-II was caused by induced autophagy or the blocked autophagy flux, we treated HEK-293T cells with Bafilomycin A1, a lysosome inhibitor that blocks autophagosome degradation. The failure of Bafilomycin A1 causing additional LC3-II accumulation in the presence of SCAMP5 (Fig 3B) indicated that SCAMP5 does not increase autophagy influx, but blocks autophagy flux.

Besides LC3-II, we examined p62, another autophagy substrate commonly used to determine autophagy activity, in both HEK-293T (Fig 3B) and SH-SY5Y cells (Fig 3C) transfected with SCAMP5 and/or treated with Bafilomycin A1. In both types of cells, SCAMP5 caused the accumulation of p62. Consistent with the pattern of LC3-II accumulation, while SCAMP5 or Bafilomycin A1 could each cause p62 buildup, the combination of both did not lead to higher p62 level (Fig 3B), indicating a blockage of autophagy by SCAMP5.

To evaluate the role of SCAMP5 in autophagy flux, we silenced the endogenous SCAMP5 and examine the accumulation of LC3II and p62 in the absence or the presence of Rapamycin (Fig 3D). Knockdown of SCAMP5 reduced the accumulation of LC3II and p62, thus further indicating the inhibitory effect of SCAMP5 in the autophagy flux.

Finally, we used the acid-sensitive mRFP-GFP-LC3 [23] as an autophagy reporter to validate the inhibition of autophagosome- lysosome fusion by SCAMP5 (Fig 3E). The acid sensitive GFP in this reporter would lose the green fluorescence upon the fusion of autophagosome with acidic lysosome. Therefore, yellow dots (RFP+GFP+) indicate autophagosome before fusion and red dots (RFP+GFP-) mark degradational auto-lysosome. Quantification of the immunofluorescence microscopy analysis indicated that SCAMP5 increased the number of autophagosomes and decreased the number of autolysosomes, especially when autophagy was augmented by starvation (Fig 3E), confirming that SCAMP5 blocked autophagy.

### SCAMP5 facilitates α-synuclein secretion upon protein stress

**Fig 4:**
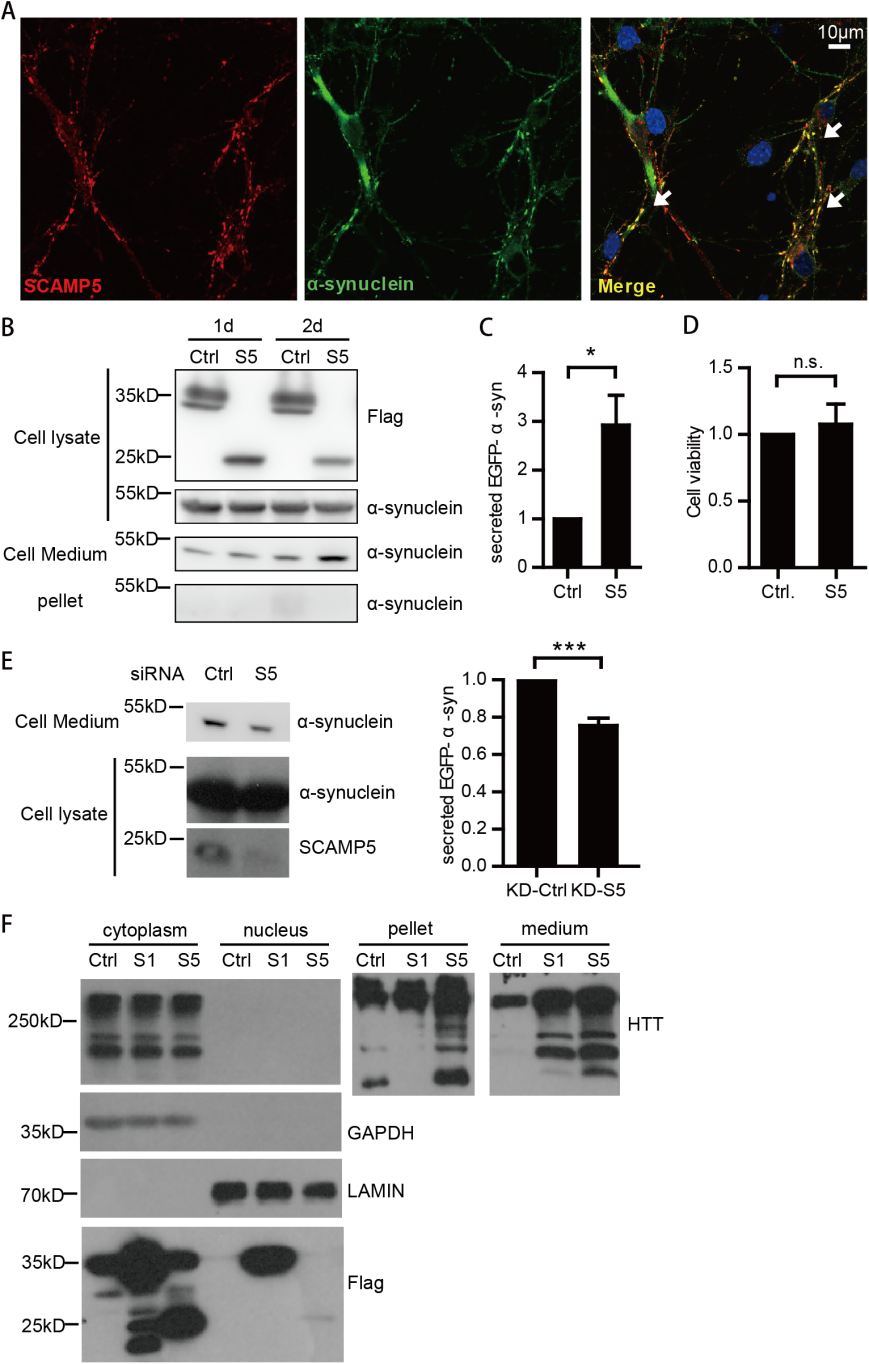
SCAMP5 facilitates the secretion of α-synuclein and huntingtin protein. (A) Immunofluorescent images of rat primary neuronal culture showing the co-localization of endogenous SCAMP5 (red) and α-synuclein (green) using confocal microscopy. (B) SCAMP5 increases the secretion of α-synuclein over time in cultured cells. EGFP-α-synuclein stable overexpressed SH-SY5Y cells were transfected with Flag-GFP or SCAMP5, and harvested 1 to 2 days after transfection. The amount of transfected EGFP-α-synuclein in cell lysate(CL), cell medium(CM), and SDS-insoluble pellet were analyzed by immunoblotting. (C) Quantification of secreted EGFP-α-synuclein in cell medium of SH-SY5Y cells overexpressed with SCAMP5 or control for 24 hours in three independent experiments. (mean ±S.E.M.; n = 3; *p<0.05). (D) MTS assay showing no significant difference in cell viability between control plasmid or SCAMP5 overexpressed cells. SH-SY5Y cells stably expressing EGFP-α-synuclein-WT were transfected with control plasmid or Flag-SCAMP5. 48 hours later, cells were digested, counted, and adjusted to the same concentration, then applied to MTS assay (mean ±S.E.M.; n = 3). (E) Knockdown of SCAMP5 decreases the secreted EGFP-α-synuclein in cell medium. SH-SY5Y cells stably expressing EGFP-α-synuclein were transfected with scrambled siRNA or SCAMP5 siRNA. The CL and CM were collected 72 hours later and examined by immunoblotting. Right panel shows the quantification of secreted EGFP-α-synuclein in cell medium of three independent experiments (mean ±S.E.M; n = 3; ***p<0.001). (F) SCAMP5 causes aggregation and secretion of HTT protein. Immunoblots showing HTT protein in cell lysates (cytoplasm and nucleus portion), RIPA-insoluble pellets (extracted with 8M Urea) and cell medium of HEK-293T cells transfected with an empty vector, SCAMP1 or SCAMP5.

As SCAMP5 suppresses autophagy and is a protein expressed at the synapse [18], we tested whether SCAMP5 may lead to the accumulation of α-synuclein, another synaptic protein whose accumulation causes Parkinson's disease[30, 31]. In primary rat cortical neurons, SCAMP5 and α-synuclein co-localize (Fig 4A). Surprisingly, in EGFP-α-synuclein over-expressing SH-SY5Y cells, SCAMP5 didn’t cause cellular accumulation of α-synuclein, there was no SDS-insoluble α-synuclein detected. Instead, SCAMP5 promoted the secretion of α-synuclein, and the amount of secreted α-synuclein in the medium gradually increased overtime (Fig 4B and 4C). The expressions of the transfected plasmids were adjusted to a modest level to ensure minimal cell death, and MTS analysis confirmed that there was no difference in cell viability between control and SCAMP5 over-expressed cells (Fig 4D). To validate the role of SCAMP5 on α-synuclein secretion, we knocked down endogenous SCAMP5 in SH-SY5Y cells, and found the reduction of secreted α-synuclein in cells with SCAMP5 depletion (Fig 4E). Previously, SCAMP5 was shown to cause the accumulation of HTT [22]. We found that SCAMP5 can not only cause the accumulation of insoluble HTT, but also promoted the secretion of HTT. In contrast, SCAMP1 did not inhibit autophagy, and only facilitated the secretion of HTT (Figs 3A and 4F). Therefore, SCAMP5 may modulate the protein aggregation and clearance pathways by affecting both autophagy and secretion, and promote secretion as the mainly clearance pathway.

### SCAMP5 causes Golgi fragmentation, and facilitates unconventional secretion of α-synuclein via the exosome pathway

**Fig 5:**
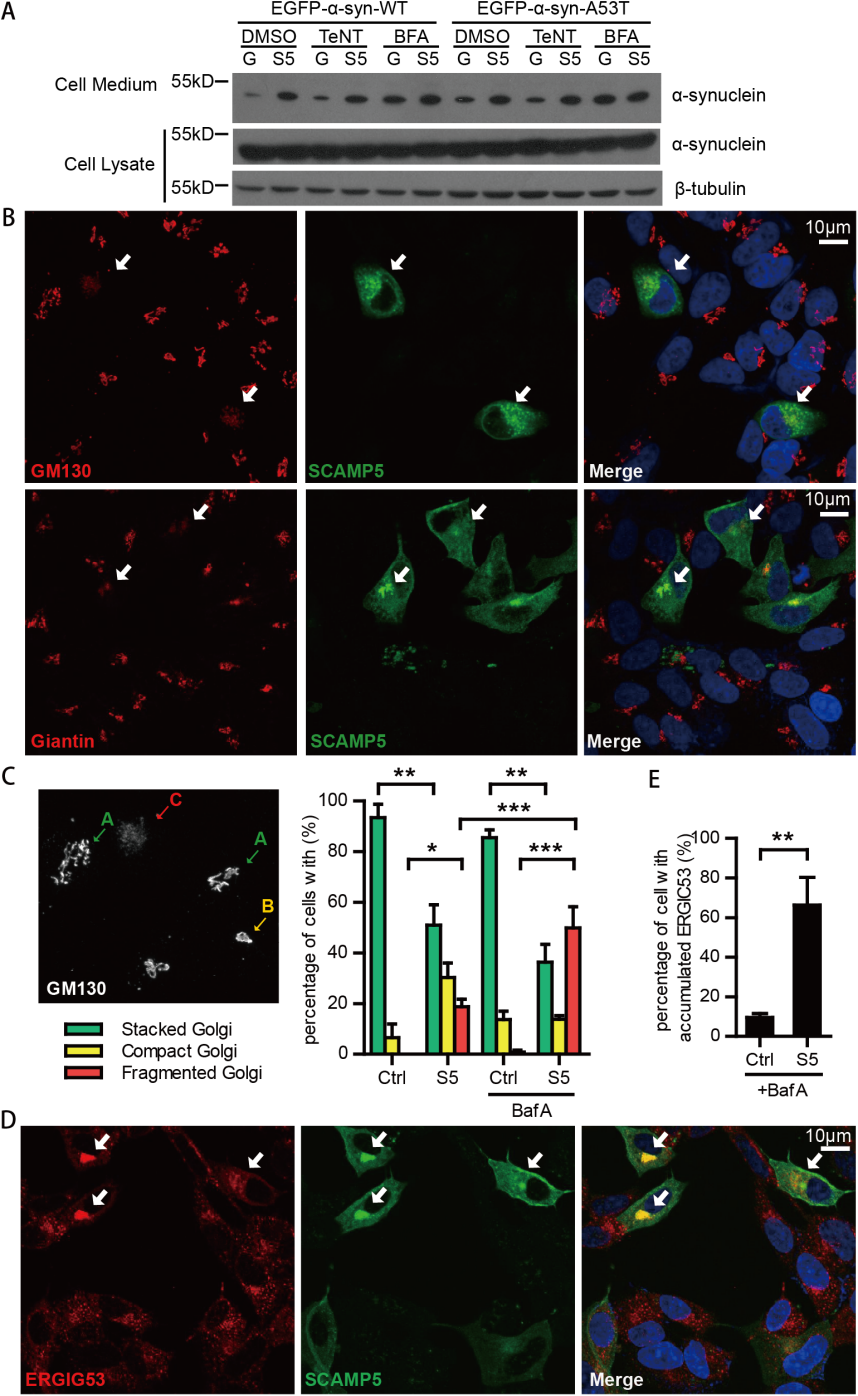
SCAMP5 facilitates the secretion of α-synuclein through Golgi-independent secretion pathway, and causes Golgi fragmentation. (A) Inhibitors of Golgi-dependent conventional secretion did not block SCAMP5-mediated secretion of α-synuclein. SH-SY5Y cells stably expressing EGFP-α-synuclein-WT or EGFP-α-synuclein-A53T were transfected with Flag-GFP or Flag-SCAMP5. Cell medium and cell lysate were harvested 48 hours later. Six hours before harvested, the exocytosis inhibitor Tetanus toxic (TeNT, 2nM) or BrefeldinA (BFA, 2μM), or DMSO were applied with a medium change. (B) Representative immunofluorescent images (z-projection) showing Golgi fragmentation in SCAMP5 over-expressed SH-SY5Y cells. Cells were transfected with Flag-SCAMP5, and treated with or without Bafilomycin A1 for 6 hours. 48 hours later, cells were fixed and immuno-stained with antibodies against Flag (green) and Golgi marker GM130 (red) or Giantin (red), and examined with confocal microscopy. Arrows indicate fragmented Golgi apparatus. (C) Quantificantion of Golgi fragmentation. The Golgi apparatus were immuno-stained with GM130 antibody and categorized into three types according to the integrity of its structure: normally stacked Golgi (green arrow), slightly collapsed compact Golgi (yellow arrow), and fragmented Golgi (red arrow). The percentages of cells with indicated Golgi morphology in SCAMP5-/+ cells were determined in three independent experiments (total cell number≥300; mean ±S.E.M.; *p<0.05, **p<0.01, ***p<0.001). (D) Immunofluorescent images showing impaired ER-Golgi transport in SCAMP5 over-expressed SH-SY5Y cells. Cells were transfected with Flag-SCAMP5 and immuno-stained with antibodies against Flag (green) and ERGIC53 (red). Representative morphology of accumulated (arrow) ER-Golgi intermediate compartment was as showed. (E) Statistics shows the percentage of cells with impaired ER-Golgi transport in SCAMP5- or SCAMP5+ cells treated with BafilomycinA1 in four independent experiments (total cell number≥300; mean ±S.E.M.; **p<0.01).

To test whether SCAMP5 stimulated α-synuclein secretion via the conventional Golgi-dependent pathway, we treated cells with Tetanus toxic(TeNT) or Brefeldin A, inhibitors for Golgi-dependent secretion[32, 33], and examined the effect of SCAMP5 on the secretion of EGFP-α-synuclein (WT and A53T). Tetanus toxin or Brefeldin A did not inhibit the SCAMP5-facilitated secretion (Fig 5A), suggesting that SCAMP5 may stimulate the secretion of α-synuclein via a non-traditional pathway. Furthermore, Brefeldin A increased α-synuclein secretion independent of SCAMP5, and the combination of Brefeldin A and SCAMP5 did not increase α-synuclein secretion further (Fig 5A), suggesting that these two factors may act in a common pathway. Since Brefeldin A can cause Golgi fragmentation [32], we evaluated whether SCAMP5 and Brefeldin A may have a similar effect on Golgi disassembly. Using specific Golgi markers GM130 and Giantin, we found that the expression of SCAMP5 caused fragmentation of Golgi apparatus (Fig 5B). Quantitative analysis indicated clear increase of fragmented Golgi and the reduction of stacked Golgi in cells expressing SCAMP5 (Fig 5C). When we enhanced the protein stress by treating the cells with lysosome inhibitor Bafilomycin A1, the percentage of fragmented Golgi significantly increased in the presence of SCAMP5. In contrast, Bafilomycin A1 alone barely caused Golgi fragmentation (Fig 5C). In SCAMP5 over-expressing cells, we also found the accumulation of ERGIC-53, a type I integral membrane protein recycled between endoplasmic reticulum and the Golgi, indicating a blockage of the secretory pathway (Fig 5D and 5E). Collectively, these results suggest that SCAMP5 does not facilitate the ER-Golgi-dependent conventional secretion. In contrast, it blocks the ER-Golgi protein trafficking and promotes the Golgi disassembly.

**Fig 6:**
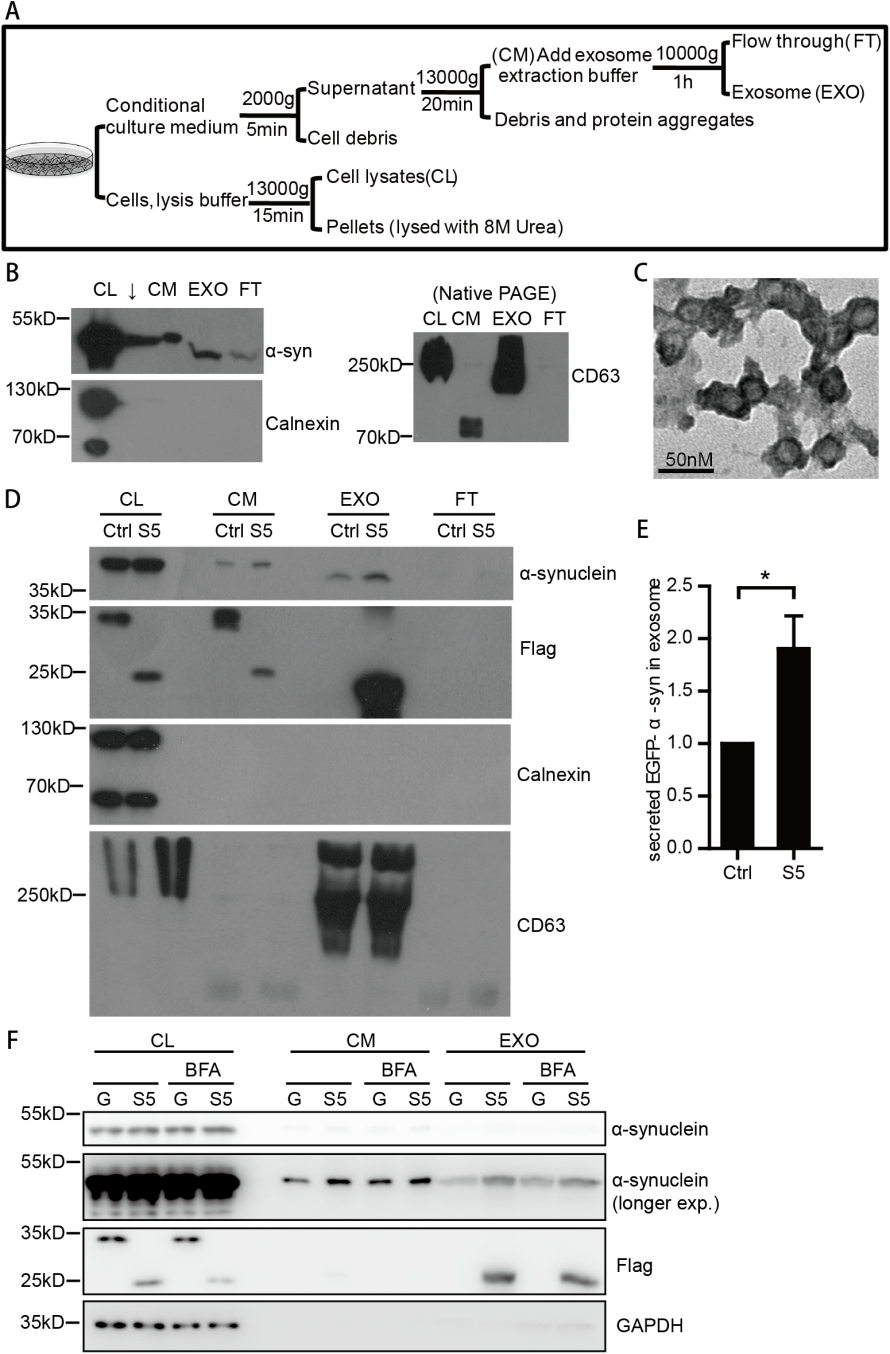
SCAMP5 facilitates the secretion of α-synuclein through exosome pathway. (A) Flowchart showing the exosome extraction procedure. (B) Quality control of exosome extraction. Cell lysates(CL), Cell medium(CM), exosome(EXO), Flow through(FT), and RIPA insoluable pellets of EGFP-α-synuclein stable overexpressed SH-SY5Y cells were collected according to the flowchart in 6A. The difference in apparent molecular weight was mainly due to different buffer between samples. Immunoblotting of exosome marker CD63 is conducted using native lysis buffer and native PAGE. Other proteins such as α-synuclein and ER marker Calnexin were examined with SDS-PAGE. (C) Electron microscopy images of exosomes isolated from the cell medium of SH-SY5Y cells stably expressing EGFP-α-synuclein. (D) SCAMP5 is abundant in exosomes, and it increases α-synuclein secretion via exosome. SH-SY5Y cells stably expressing EGFP-α-synuclein were transfected with Flag-GFP or Flag-SCAMP5, and harvested 48 hour later. All the samples were processed identically. The loading/total volumes of CL, CM, EXO, and FT were 2/1,200μL, 30/6,000μL, 30/200μL, 30/6,000μL respectively. (E) Quantification of secreted EGFP-α-synuclein in exosome of SH-SY5Y cells overexpressed with SCAMP5 or control in three independent experiments. (mean ±S.E.M.; n = 3; *p<0.05). (F) SH-SY5Y cells stably expressing EGFP-α-synuclein were transfected with Flag-GFP or Flag-SCAMP5, and harvested 48 hour later. BrefeldinA was added 6 hours before harvest with a change of medium.

Since α-synuclein could be secreted through exosome, which is an unconventional secretion pathway [16], we tested whether SCAMP5 facilitated the secretion of α-synuclein via exosome. The exosomes in the culture medium of EGFP-α-synuclein stable SH-SY5Y cells were isolated (Fig 6A), and the quality of the isolation was confirmed using exosome marker CD63 and morphorlogical criteria (Fig 6B and 6C), endoplasmic reticulum(ER) marker Calnexin was used as a control for cellular content. SCAMP5 clearly increased the abundance of secreted α-synuclein via exosomes (Fig 6D and 6E). BrefeldinA, which also causes Golgi fragmentation and inhibits conventional secretion, had similar effect on α-synuclein secretion as SCAMP5 (Fig 6F). Moreover, SCAMP5 was readily detectable in exosomes, indicating that SCAMP5 not only facilitated exosome secretion, but was also secreted through exosomes (Fig 6D). Therefore, besides being degraded via proteasome and autophagy pathway (Fig 2D and 2E), SCAMP5 was also cleared by secretion via exosome. Therefore, these results demonstrated that SCAMP5 is enriched in exosomes and may act as a carrier to facilitate the exosome-mediated clearance of α-synuclein.

## Discussion

In this study, we have reported SCAMP5 as a novel regulator of protein clearance during stress. SCAMP5 facilitates the clearance of neurotoxic proteins, such as α-synuclein, in a seemingly paradoxical manner. SCAMP5 is quickly induced by autophagy signals, but inhibits autophagy by blocking the fusion of autophagosome and lysosome. On the other hand, SCAMP5 expression leads to increased Golgi fragmentation and promotes the secretion of α-synuclein via exosomes. The transient induction of SCAMP5 upon protein stress suggests a cellular mechanism whereby SCAMP5 is produced on demand to quickly remove accumulated proteins in the most efficient way, by promoting secretion.

Nevertheless, this cellular protective protein release mechanism may initiate the spreading of neurotoxic proteins. The propagation of neurotoxic proteins has been gradually accepted as a new mechanism leading to the development of neurodegenerative diseases [9, 34–36]. The intercellular spreading of Aβ, Tau, α-synuclein, HTT, TDP43 and C9ORF72 dipeptide repeats have been documented[15, 37–40]. However, the trigging events and the regulators involved in the process of protein propagation are not clear. Our study described SCAMP5 as an inducible regulator linking several aspects of the protein clearance pathway. First, SCAMP5 is induced under protein stress conditions at the transcription level by the master autophagy transcriptional regulator TFEB. Next, SCAMP5 itself could be normally degraded by both the proteasome and autophagy machinery, as inhibition of either pathway lead to a transient increase of the SCAMP5 protein. Third, the increase of SCAMP5 inhibits autophagy but promotes the secretion of neurotoxic proteins including α-synuclein and HTT. Finally, SCAMP5 increases Golgi fragmentation and facilitates the non-conventional exosome secretion. Under normal conditions, autophagosomes can be fused with endocytic structures such as MVBs to generate amphisomes, then fused with lysosomes to degrade the contents [41]. SCAMP5 blocks the fusion between autophagosome and lysosomes, and facilitates the secretion of MVB content exosomes in a non-conventional secretion pathway. Therefore, the induction of this synaptic protein serves a special purpose to guide the quick clearance of unwanted protein via exosomes. Unlike SCAMP5, a closely related SCAMP1 in the same protein family, did not inhibit autophagy.

Recent evidence has pointed to exosome-mediated unconventional secretion as a mean to increase the clearance of neurotoxic proteins[15, 34, 36], and the involvement of autophagy suppression in the process[16, 42, 43]. For example, α-synuclein, TDP-43 and HTT are secreted via exosomes[15, 35, 38]. Consistent with our finding, Poehler et al have reported increased secretion of α-synuclein when the autophagy-lysosomal pathway is inhibited [16]. Our results extend these findings, and describe α-synuclein-colocalizing SCAMP5 as a novel autophagy inhibitor and carrier of exosome-mediated secretion during protein stress. Furthermore, our findings suggest that the induction of SCAMP5 could act as a marker for accelerated exosome-mediated secretion leading to the propagation of neurotoxic proteins.
